# Regulation of non-conventional therapies in Portugal: lessons learnt for strengthening human resources in health

**DOI:** 10.1186/s12960-021-00655-3

**Published:** 2021-09-17

**Authors:** Pascoal Amaral, Inês Fronteira

**Affiliations:** 1 Escola de Medicina Tradicional Chinesa de Lisboa, Lisbon, Portugal; 2 Global Health and Tropical Medicine, Instituto de Higiene e Medicina Tropical, Universidade Nova de Lisboa, Rua da Junqueira, 100, 1359-008 Lisbon, Portugal

**Keywords:** Non-conventional therapies, Regulation, Workforce, Portugal, European Union

## Abstract

**Background:**

The integration of non-conventional therapies (NCT) into health policies and health services delivery is a worldwide trend and might have a role in achieving Universal Health Coverage. WHO has encouraged countries to integrate NCT into health service delivery and to increase the interest and utilization by consumers. Following two resolutions by the European Parliament and by the Council of Europe, in the late 1990s, recommending the recognition of NCT and calling for EU legislation on non-conventional forms of medicine, Portugal initiated, in 2003, its path towards regulation of NCT. We analyze this process and discuss its implications and impacts in terms of health policies, health services delivery and overall health workforce.

**Case presentation:**

The need to regulate NCT in Portugal stemmed from a growing demand for NCT (and acceptability) among lay citizens and a positive attitude among conventional health professionals which also advocated for a regulatory framework. Political efforts undertaken since 2003 allowed for important advances in the regulation of NCT, beneficiating safe professional practices, and ensuring future academic training at the highest standards, with the defining moment of the social and legal model transition occurring in 2013, when acupuncture, chiropractic, homeopathy, naturopathy, osteopathy, phytotherapy and traditional Chinese Medicine were recognized and regulated. Nevertheless, and because the process knew important time gaps, significant deficiencies arose, mainly between regulation of the training and of the professional activities and the capacity to ensure the continuous production of NCT professionals at an acceptable rate and with minimum quality standards guaranteed.

**Conclusions:**

The regulation of NCT in Portugal was lengthy but steady and was able to bring consumers a safer practice environment and NCT professionals a legal and deontological umbrella for their training, practice, and professional development.

Nevertheless, and despite the growing acceptability and normative quality assurance of NCT and its workforce, the regulation process has highlighted some fragilities in terms of accessibility and availability that need attention and urgent action to achieve universal coverage.

## Background

The World Health Organization (WHO) has recognized the distinct nature and scope of practice of non-conventional therapies (NCT) as well as their potential to extend the diagnostic and intervention capacity of conventional medicine in both developed and developing countries [1].

All over the world, the integration of NCT into health policies and health services delivery has been trending with some authors stressing out the role of NCT in achieving Universal Health Coverage [2–4] and in contributing to the quality, efficiency, equity, accountability, sustainability and resilience of the health system. As so, WHO has encouraged countries to integrate NCT into health service delivery and to increase the interest and utilization by consumers [3].

One of the paths towards integration of NCT is through regulation of NCT professionals, services, and products as well as education and training. Through the definition of who practices the profession, what are the boundaries in which professionals operate and what are the requirements, both in terms of professional qualifications and services, to practice, the primary purpose of regulation is to protect the public from unqualified practitioners and malpractice [5].

In the 1990s, in the face of a growing and unregulated demand for NCT [6] (it was estimated that around 30–50% of citizens used it), the European Union (EU) recognized that *“different methods of treatment and different approaches to health and illness are not mutually exclusive, but can on the contrary be used to complement one another”* and that *“it* [was] *important to ensure that patients have the broadest possible choice of therapy, guaranteeing them the maximum level of safety and the most accurate information possible on the safety, quality, effectiveness and possible risks of so-called non-conventional medicines, and that they are protected against unqualified individuals”* [7]. At the time, EU countries were divided between those for which the practice of medicine by other professionals besides medical doctors was illegal (southern countries, France, Belgium and Luxembourg) and those who understood that only certain activities were strictly reserved for doctors and that these professionals were the authorities and point of reference for the health system (e.g., United Kingdom, Ireland, Netherlands, Germany, Denmark, Sweden and Finland) [7].

In 1998, several European countries participated in a research to *“demonstrate the possibilities, limitations and significance of complementary/alternative medicine by establishing a common scientific background, helping to control health care costs, and harmonizing legislation”* [8].

In Portugal, and despite the growing demand for NCT, there was no regulatory or legal framework that ensured protection and promotion of the needs of the clients and that the four key principles for provision of health care were met (availability, accessibility, acceptability, and quality) [9]. There were no boundaries on the operations of NCT services or professionals, including requirements and qualifications to practice.

Following two resolutions by the European Parliament and by the Council of Europe, in the late 1990s [10, 11], recommending the recognition of NCT and calling for EU legislation on non-conventional forms of medicine, Portugal initiated, in 2003, its path towards regulation of NCT. In this paper, we analyze this process in terms of regulation of practice, education, and professional development, and discuss its implications and impacts in terms of health policies, health services delivery and overall health workforce.

## Case presentation

The regulation of NCT in Portugal began in 2003 with a resolution from the Parliament recommending the Government to study the type of structure and method to regulate the organization, deontology and teaching of osteopathy, and to create a commission to certify the national courses and accredit the professionals trained abroad [12].

### Formal recognition and definition of NCT in the Portuguese context

In 2003, a law on the basic framework of the NCT [13] was published recognizing acupuncture, homeopathy, naturopathy, phytotherapy, and chiropractic as NCT. NCT were defined as having a different philosophical background from medicine, with specific diagnosis and therapeutical processes and to be pursued by qualified, accountable and competent professionals [13]. Technical and deontological autonomy were recognized and the tutelage of NCT was given to the Ministry of Health (similar to other health professions not self-regulated organized in professional councils). It was also defined that the practice of NCT, regardless of the area, could only be carried out by legally qualified and accredited professionals. A technical commission was created to study and propose the general framework for regulating the exercise of NCT until 2005, when the process of regulating the credentialing, training, and certification of professionals was expected to be completed [13].

However, the deadlines decreed were not met and there was an interregnum of more than 8 years before any further legislative action was taken. As a consequence, in 2011, the Parliament recommended the Government to urgently resume the regulation of NCT [14].

### Practice

Finally, in 2013, the 2003 Law was regulated and Traditional Chinese Medicine (TCM), not previously included, was added [15]. The terms for assessing NCT professions were defined (i.e., bachelor with honors), and the Central Administration of the Health System *“in charge of managing financial and human resources, facilities and equipment, systems and information technology of the National Health System”* [16], became responsible for issuing professional licenses. A grandfather clause was included in the law for those professionals already practicing NCT. These professionals had, within 180 days from the publication of the law, to require formal recognition to ACSS of their suitability to provide NCT care based on: proof of practice; curriculum vitae; in-service training, and professional development; experience in the administration of pharmaceutical products. Until 2021, this clause was resumed several times to accommodate NCT practitioners that had not regularized their professional status.

As a consequence, in 2014, a working group for the curricular evaluation of NCT professionals was created to assess applications for professional licenses from existing NCT professionals. The curricular evaluation was based in a system of points that comprehended education, professional experience, additional training in the area of practice and internships [17]^18^.

Accountability of professional activity was foreseeing by the obligation of all NCT professionals to have a professional insurance. The entities responsible for inspection and control of NCT activities were defined, with the main one being the Inspectorate General of Health-related Activities, as well as the sanctions regime [15].

Still in 2014, the minimum standards for NCT clinics, in terms of infrastructures, equipment and human resources were defined and became subjected to the same regulations that other clinical practices by the Health Regulatory Agency [17].

### Education and training

In 2014, the Ministry of Health and the Ministry of Higher Education, in accordance with the WHO recommendations, published seven ordinances defining the scope of practice and deontological obligations of the acupuncturist [19], the TCM specialist [20], the herbalist [21], the homeopath [22], the naturopath [23], the osteopath [24], and the chiropractor [25].

This led to the joint definition, by the Ministry of Health and the Ministry of Higher Education, in 2015, of the general training requirements for acupuncture [26], phytotherapy [27], naturopathy [28], osteopathy [29] and chiropractic [30] and two and a half years later, in 2018, for TCM [31]. All required a four-year, university degree (bachelor with honors—*licenciatura*). As a consequence, professional licenses to practice any of the NCT were subject to the requirement of having a university degree. However, in 2019, an amendment was made to modify the regime for granting professional licenses [32]. It was defined that until December 31, 2025, or until the awarding of the first degree in each of the regulated NCT, it would be possible to get the professional license for those who completed their training in a non-higher education institution.

This amendment, aimed exclusively at students who finished their education after October 2, 2013, came as a result of a recommendation from the Parliament to the Government to ensure equity in the access to the profession for those who, after 2013 and with no alternative higher education training, continued to carry out their training in institutions not integrated in the higher education system [33].

The teaching of NCT courses began to be regulated by the Ministry of Higher Education, in 2015. This meant that training programs had now to be accredited by the Agency for Accreditation of Higher Education, thus fulfilling the exact same requirements of other training programs, including those pertaining to other health professionals, like doctors or nurses. However, and up until 2021, no similar requirements were established for homeopathy, which remains quite controversial, especially among some groups of health professionals.

As a result of the regulation of the training courses in NCT areas, in 2016, five NCT study cycles in osteopathy were created within the higher education system, with the first two being created in the private sector, one in the North and one in the South of the country [34]^35^. The subsequent years saw a rise in the number of accredited NCT training programs in both the private and the public sector, more or less spread throughout the country, but only in acupuncture and osteopathy. For the remaining regulated NCT, the training offers remained outside the higher education system with no training program so far accredited. In 2021, there were eight higher education programs accredited for osteopathy and 3 for acupuncture [36] (Box 1). In 2020, the first osteopathy degrees were awarded, and in 2021 it is expected that the first acupuncturists graduate (Box 1).

**Box 1:** NTC facts and figure 2021 [35, 53]

7 NTC regulated with definition of scope of practice (chiropractic, acupuncture, homeopathy, TCM, phytotherapy, naturopathy, osteopathy)
6 out of 7 with training requirements approved (except for homeopathy) 6160 NTC professionals registered and authorized to practice: 1482 in acupuncture, 511 in phytotherapy, 620 in naturopathy, none in homeopathy,
2091 in osteopathy, 1410 in TCM, and 46 in chiropractic
59.8 NTC professionals per 100 000 inhab
8 higher education training programs in osteopathy and 3 in acupuncture: 210 vacancies open in 2020/2021 school year for osteopathy and 103 vacancies for acupuncture
Phytotherapy, naturopathy, TCM, homeopathy and chiropractic not offered at university level

### Continuing to develop a legal regulatory framework for NCT

**Fig. 1:**
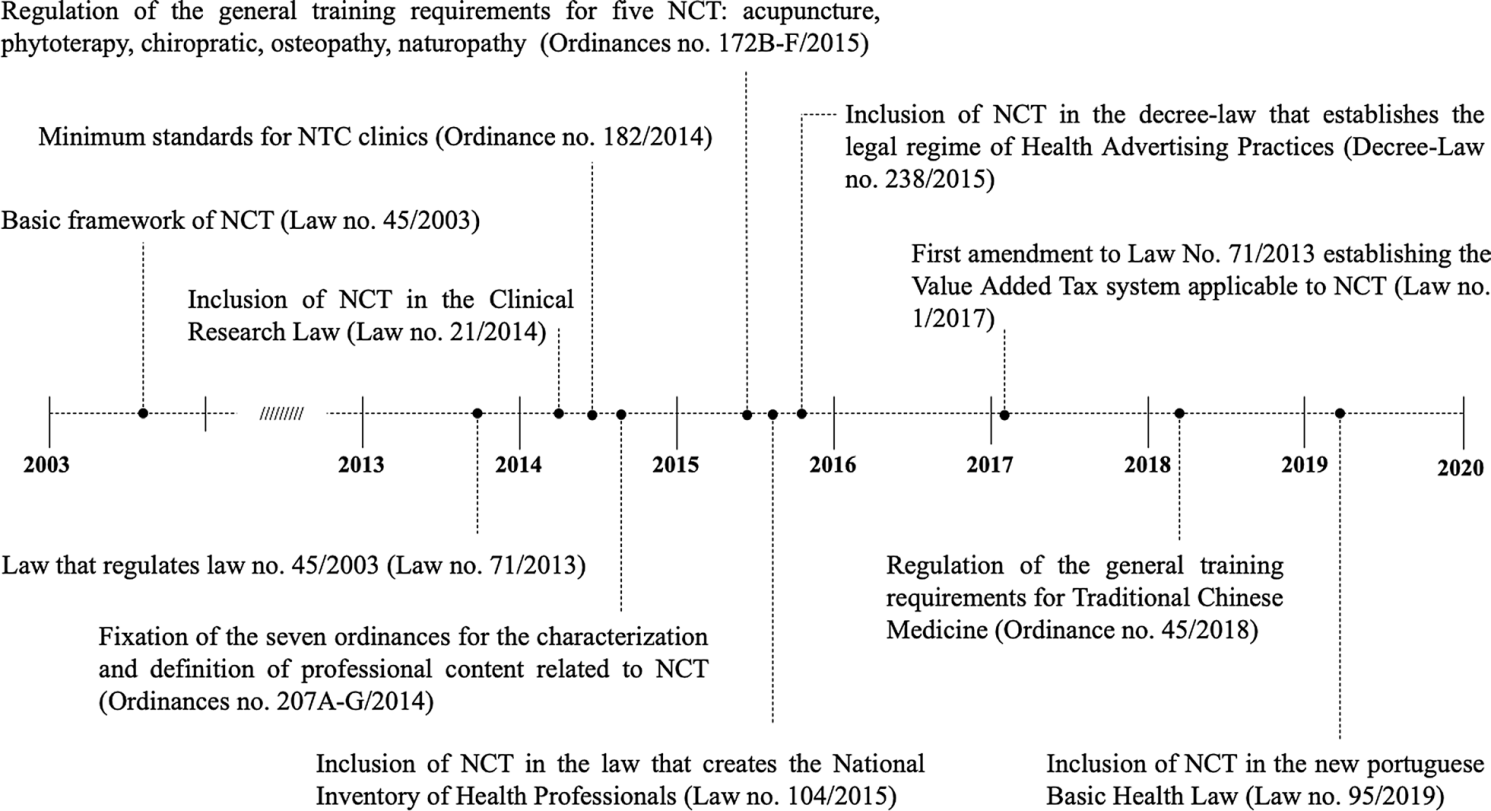
Timeline of the regulation of NCT in Portugal

Since 2014, NCT have been progressively included in other juridical pieces pertaining to health (e.g., 2014 law on clinical research with inclusion of NCT trials; inclusion, in 2015, in the law that created the National Inventory of Health Professionals) [37], 38 (Fig. 1).

Finally, in the 3rd quarter of 2019, a new Health Basic Law was published, where NCT found base 26 where it is stated that NCT is *“carried out in an integrated manner with conventional therapies and in order to guarantee the protection of people's and communities' health, the quality of care, and based on the best scientific evidence”*[39].

### Current situation on NCT professionals in the Portuguese health system

To date, 6160 NCT professional licenses have been issued by ACSS (1482 in acupuncture, 511 in phytotherapy, 1410 in TCM, 620 in naturopathy, 2091 in osteopathy and 46 in chiropractic) [40], which corresponds to a ratio of 59.8 NCT professionals per 100 000 inhabitants. Due to the delay in defining the training program for homeopathy, no professional license in homeopathy has been issued so far. All homeopaths are thus, practicing without any regulation.

## Discussion and conclusions

The regulation of NCT in Portugal, that culminated with the recognition of NCT as one of the bases of the health system in the 2019 Health Basic Law, was lengthy, unstable, and discontinuous, and one dares to say more reactive than proactive, which impaired its contribution towards UHC.

Nowadays the Portuguese Health System is transitioning between an inclusive, and an integrative system based on the relationship between allopathic and traditional/complementary medicine. Despite being regulated and recognized, with education being partially available, NCT are not available at NHS hospitals and clinics, and treatments are not reimbursed under health insurance [41]. Consequently, and so far, the regulation of NCT has contributed to that which is the main role of regulation—protect the client—but it is far to contribute to increased access to NCT or coverage. NCT are mainly used by a marginal fringe of the Portuguese population that tend to be urban, well-educated and middle-class.

Nevertheless, the political efforts undertaken since 2003 are remarkable and allowed for important advances in the regulation of NCT, benefitting safe professional practices, and ensuring future academic training at the highest standards, with the defining moment of the social and legal model transition occurring in 2013, when acupuncture, chiropractic, homeopathy, naturopathy, osteopathy, phytotherapy and TCM were recognized and regulated. Nevertheless, because the process knew important time gaps, significant deficiencies arose, mainly between regulation of the training and of the professional activities and the capacity to ensure the continuous production of NCT professionals at an acceptable rate and with minimum quality standards guaranteed.

The need to regulate NCT stemmed from a growing demand for NCT (and acceptability) among lay citizens and a positive attitude among conventional health professionals who also advocated for a regulatory framework on the same deontological and legal grounds as theirs [42–46]. A similar trend was observed in other countries with osteopathy frequently leading the way [47].

Portugal seems to be on a convergent path towards regulation of NCT when compared to other European countries. According to 2012 data Belgium, Denmark, Germany, Iceland, Liechtenstein, Norway, Hungary, Slovenia, Romania and Serbia have a NCT-specific law, and some have regulated professions, recognized in the European Union: acupuncture (Malta and Switzerland), homeopathy (Switzerland), naturopathy (Switzerland), and osteopathy (Finland, Iceland, Liechtenstein, Malta, Switzerland and the United Kingdom) [48].

The base of the regulation process of NCT was always the complementary role to conventional approaches to health and included the definition of seven NCT, the minimum standards for practice, the scope of practice of each NCT, the tutelage by the Ministry of Health, the legal accountability, and the deontological framework along with the training at higher educational level. This process allowed for more than 6,000 professionals with NCT competencies to get a professional license. Nevertheless, currently, to enter any NCT regulated profession, a university degree is required. However, and despite the number of osteopathy and acupuncture training programs that, since 2016, have been accredited and are being offered at higher education level, for the remaining NCT there are no accredited training courses so far and for homeopathy the regulation of the training is still lacking, which means that no professionals in this area are being trained within the requirements to assess the profession. Additionally, transitional measures to assure that the training capacity (and consequently professional recognition) will remain at the same level, while scaling up the training level are needed. Accreditation of training programs at university level demand not only a coherent curriculum, but above all a strong demonstration that the training institution has the sufficient human resources needed (in number and quality) to deliver the training program [36]. TCM which has been taught since the 1990s [49] and is the third most used NCT in Portugal was not accredited. Because NCT professionals were traditionally trained outside universities, most of them might not qualify to attend post-graduate training (e.g., master and doctoral programs). On the other hand, the training capacity might also be jeopardized by the (low) number of existing NCT professionals that might have to either accumulate teaching and clinical activities or the choose between them. In addition to these factors, the logistical requirements and consequently the financial resources that must be available in traditional schools are huge problems that must be overcome if these schools are intended to move into the higher education system.

Although the NCT process might seem almost finished, exception made for homeopathy, soon other NCT might emerge and further regulation might be needed like, as was the case in other countries [48].

The case of homeopathy is a peculiar one. Among all NCT, homeopathy is perhaps the one that has raised more discussion not only in Portugal but also all over the world, despite the annual expenditure for its remedies and frail evidence of its effects [50, 51] which might explain the delay observed (while, for instance, medical acupuncture is a competency recognized by the medical council in Portugal) [52, 53].

Nevertheless, NCT are not free from controversies with lack of scientific evidence being one of the main arguments for their opposers. However, if one thinks of NCT as complementary to conventional therapies, and not an alternative, NCT can and should be strengthened to bring a more holistic approach not only to disease or ill-health states but, above all, to promotion of well-being.

The future challenges for NCT in Portugal are now different. The inclusion of NCT as a base of the health system as raised the question (and sooner or later the debate) if NCT are going to be integrated in the National Health Service and, if so, on what grounds. At its current state, access to NCT is not universal. NCT are provided only by the private sector with the costs being supported exclusively by the user, even for those covered by voluntary health insurance or a health subsystem.

As demand is expected to grow, especially if Portugal follows the international trend, there might be a bottleneck in increasing the availability of the NCT workforce, namely in terms of the training capacity. The recent scale-up of the training of NCT professionals to the university level has raised issues related to quality and availability of the teaching body and of the existing training institutions.

In sum, the regulation of NCT in Portugal stressed out that the need for a sector wide approach, with a combined top-to-bottom legal framework and a bottom-up implementation. Laws are important to legally frame and protect the public and the professionals, but they need to be considered in the broader context (i.e., educational and health systems) to assure sustainability.

Regulation of NCT in Portugal was lengthy but steady and was able to bring consumers a safer practice environment and NCT professionals a legal and deontological umbrella for their training, practice and professional development. Nevertheless, and despite the growing acceptability and normative quality assurance of NCT and its workforce, the regulation process has highlighted some fragilities in terms of accessibility and availability that need attention and urgent action to achieve universal coverage. The way forward is towards an integrative Portuguese Health system, where anyone can access NCT in complement with the conventional provision of care.

## Data Availability

Not applicable.

## Abbreviations

NCT: Non-conventional therapies
EU: European Union
TCM: Traditional Chinese medicine
WHO: World Health Organization
